# Experimental and molecular approximation to microbial niche: trophic interactions between oribatid mites and microfungi in an oligotrophic freshwater system

**DOI:** 10.7717/peerj.5200

**Published:** 2018-07-11

**Authors:** Patricia Velez, Margarita Ojeda, Laura Espinosa-Asuar, Tila M. Pérez, Luis E. Eguiarte, Valeria Souza

**Affiliations:** 1 Departamento de Botánica, Instituto de Biología, Universidad Nacional Autónoma de México, Mexico City, Mexico; 2 Colección Nacional de Ácaros, Departamento de Zoología, Instituto de Biología, Universidad Nacional Autónoma de México, Mexico City, Mexico; 3 Departamento de Ecología Evolutiva, Instituto de Ecología, Universidad Nacional Autónoma de México, Mexico City, Mexico

**Keywords:** Mites-fungal interactions, Oligotrophic system, Oribatei, Aspergillus, Mite dietary preferences, Trhypochthoniidae

## Abstract

Mite-fungal interactions play a key role in structuring core ecosystem processes such as nutrient dynamics. Despite their ecological relevance, these cross-kingdom interactions remain poorly understood particularly in extreme environments. Herein, we investigated feeding preferences of a novel genetic lineage of aquatic oribatids obtained from an oligotrophic freshwater system in the Cuatro Ciénegas Basin (CCB) within the Chihuahuan Desert, Mexico. During in vitro diet preference bioassays, transient aquatic microfungi (*Aspergillus niger*, *Talaromyces* sp., and *Pleosporales* sp.) recovered from the same mesocosm samples were offered individually and simultaneously to mites. Gut content was analyzed using classic plating and culture-independent direct PCR (focusing on the fungal barcoding region) methods. Our results indicated that oribatids fed on all tested fungal isolates, yet the profusely developing *A. niger* was preferentially consumed with all fungal components being digested. This feeding habit is particularly interesting since *A. niger* has been reported as an unsuitable dietary element for population growth, being consistently avoided by mites in previous laboratory experiments. It is possible that our mites from the CCB have adapted to exploit available resources within this oligotrophic site. This work confirms the trophic relationship between microfungi and mites, two rarely investigated major components of the microbial community, shedding light on the niche dynamics under low-nutrient conditions.

## Introduction

As a keystone concept in ecology, vast literature has accumulated around the concept of the ecological niche (reviewed by Soberón, 2007). However, knowledge on the biotic component of this concept remains largely unexplored in microorganisms (some studies are available in aquatic Hyphomycetes, Arsuffi & Suberkropp, 1984; Chauvet et al., 2016; Duarte et al., 2017), as most of this work has been concentrated on vertebrates and angiosperms. Extending this framework to microbes is relevant as these organisms represent the vast majority of the taxonomic, genetic and metabolic diversity and biomass on the planet.

As key components of the microscopic world, microfungi and mites represent large and diverse elements in natural communities, fulfilling numerous ecological roles (Kjøller & Struwe, 1982; Dighton, 1997; Maraun & Scheu, 2000). Microfungi are involved in the decomposition of organic matter and nutrient cycling (Scheu, 1993), degrading highly recalcitrant organic compounds such as lignin (Dijkstra, Boon & Van Mourik, 1998). Mites, for their part, are a dominant group of soil invertebrates (Strenzke, 1952; Schatz, 2002) that play an active role in litter decomposition (Wallwork, 1970), vertical translocation of organic matter for deeper soil (Wallwork, 1967), humus formation (Kubiena, 1953) and dispersion of decomposer taxa (Healey, 1970; Harding & Stuttard, 1974).

Trophic interactions between mites and fungi are diverse and complex, affecting core ecosystem processes such as nutrient dynamics (Ingham et al., 1985; Hofstetter & Moser, 2014). Fungal grazing by mites has been associated with increased decomposition rates, nutrient cycling, and plant growth (Klironomos & Kendrick, 1995; Bonkowski et al., 2000; Gange, 2000; Cortet et al., 2002). Furthermore, changes in microbial respiration (Bengtsson & Rundgren, 1983; Kaneko et al., 1998), fungal biomass production, interactions, and distribution have also been attributed to mite grazing (Hanlon & Anderson, 1979; Visser, Whittaker & Parkinson, 1981; Lussenhop, 1992; Bardgett, Whittaker & Frankland, 1993a, 1993b; Tiunov & Scheu, 2005).

At the individual level, mite-fungal trophic associations benefit fungi by enabling selective spore dispersal (Jacot, 1930; Griffiths, Hodson & Christensen, 1959; Stefaniak & Seniczak, 1976, 1981; Williams, Whipps & Cooke, 1998a, 1998b; Meier, Scherrer & Honegger, 2002) and mites obtain valuable nutrients such as sterols and vitamins from fungal components (Van Asselt, 1999). Nevertheless, these interactions (corresponding to the niche space of each species) remain poorly understood in oligotrophic aquatic habitats.

Oribatids, a major component of taxonomic mite diversity (Walter & Proctor, 1999), have a wide-ranging diet (Schuster, 1956; Luxton, 1972) based on fungal elements (Siepel & Maaskamp, 1994). These arthropods feed selectively when high-quality food is available leading to the term “choosy generalists” (Schneider & Maraun, 2005). Furthermore, food choice experiments have shown that these arthropods prefer ubiquitous, dark pigmented fungi as dietary elements (Maraun et al., 1998; Koukol et al., 2009). However, feeding on arbuscular mycorrhizal (Lussenhop, 1992; Larsen & Jakobsen, 1996; Hopkin, 1997; Klironomos & Moutoglis, 1999; Cole et al., 2004) and ectomycorrhizal fungi has also been documented (Schneider, Renker & Maraun, 2005).

Feeding preferences in fungivore oribatid mites have been linked with their ability to digest fungal components (Bowman, 1984; Hubert, Zilova & Pekar, 2001), tolerate fungal secondary metabolite production (Daneshvar & Rodriguez, 1979), and the accessibility of alternative food sources (Parkinson et al., 1991a, 1991b). Four general fungal-based diet assemblages have been proposed for mites (Chen, Schneider & Schneider, 1995; Kaneko, Mclean & Parkinson, 1995; Sadaka-Laulan et al., 1998). These include fungi that are preferred and suitable for mite population growth (+/+) such as *Alternaria alternata*, and *Cladosporium cladosporioides*; preferred, but unsuitable (+/−) as is the case with *Aspergillus amstelodami* var. *amstelodami*; avoided, but suitable (−/+), e.g., *Aspergillus versicolor*, *Mycocladus corymbifer*, and *A. amstelodami* var*. montevidensis*; and avoided and unsuitable fungi (−/−) such as *A. niger* (Hubert et al., 2004). In addition to these classifications, feeding guilds have been defined to understand the ecological roles of oribatids (Schuster, 1956; Luxton, 1972, Wallwork, 1983; Kaneko, 1988). Guilds refer to taxa involved in: (1) comminution of organic matter, (2) alteration of microbial activity or dispersal of decomposer microorganisms (Behan & Hill, 1978; Swift, Heal & Anderson, 1979; Wallwork, 1983), and (3) ground-dwelling herbivores with no role in organic matter decomposition.

Since trophic relationships between oribatid mites and microfungi are complex and poorly characterized, particularly in aquatic and nutrient-poor systems, the aim of this work was to evaluate feeding preferences of oribatid mites from an oligotrophic freshwater oasis in the Cuatro Ciénegas Basin (CCB) in the Chihuahuan Desert. During in vitro bioassays, we offered three microfungal taxa, including an abundant fungal species (*A. niger*) that has been previously reported as avoided and unsuitable for mite development in other systems. Our findings highlight the implications of mite-fungus interactions on organic matter decomposition and nutrient cycling under nutrient-depleted conditions.

## Materials and Methods

### Study site

The CCB (26°50′N, 102°8′W) poses a biodiversity paradox, as this extremely oligotrophic oasis harbors remarkably high levels of biodiversity (Souza et al., 2006, 2012), being regarded a model for Cambrian food webs at a stoichiometric knife-edge (Elser et al., 2006). Numerous freshwater systems containing rich microbial diversity (including transient aquatic microfungi) are distributed within this area (Souza et al., 2006, 2012; Desnues et al., 2008; Velez et al., 2016). The climate in the CCB is generally hot and arid with extreme temperatures such as 45 °C in July and temperatures below 0 °C being commonly reported in January (Conagua, 2015). Specifically, the Churince freshwater system (26°50′N; 102°8′W) comprises a perennial spring and two desiccation lagoons that are connected by short shallow streams (López-Lozano et al., 2012). This endangered system exhibits extreme and fluctuating temperature and water chemistry conditions, characterized by high concentrations of sulfates and scarce nutriments regarded as oligotrophic (Minckley & Cole, 1968; Tobler & Carson, 2010).

### Sampling

**Figure 1:**
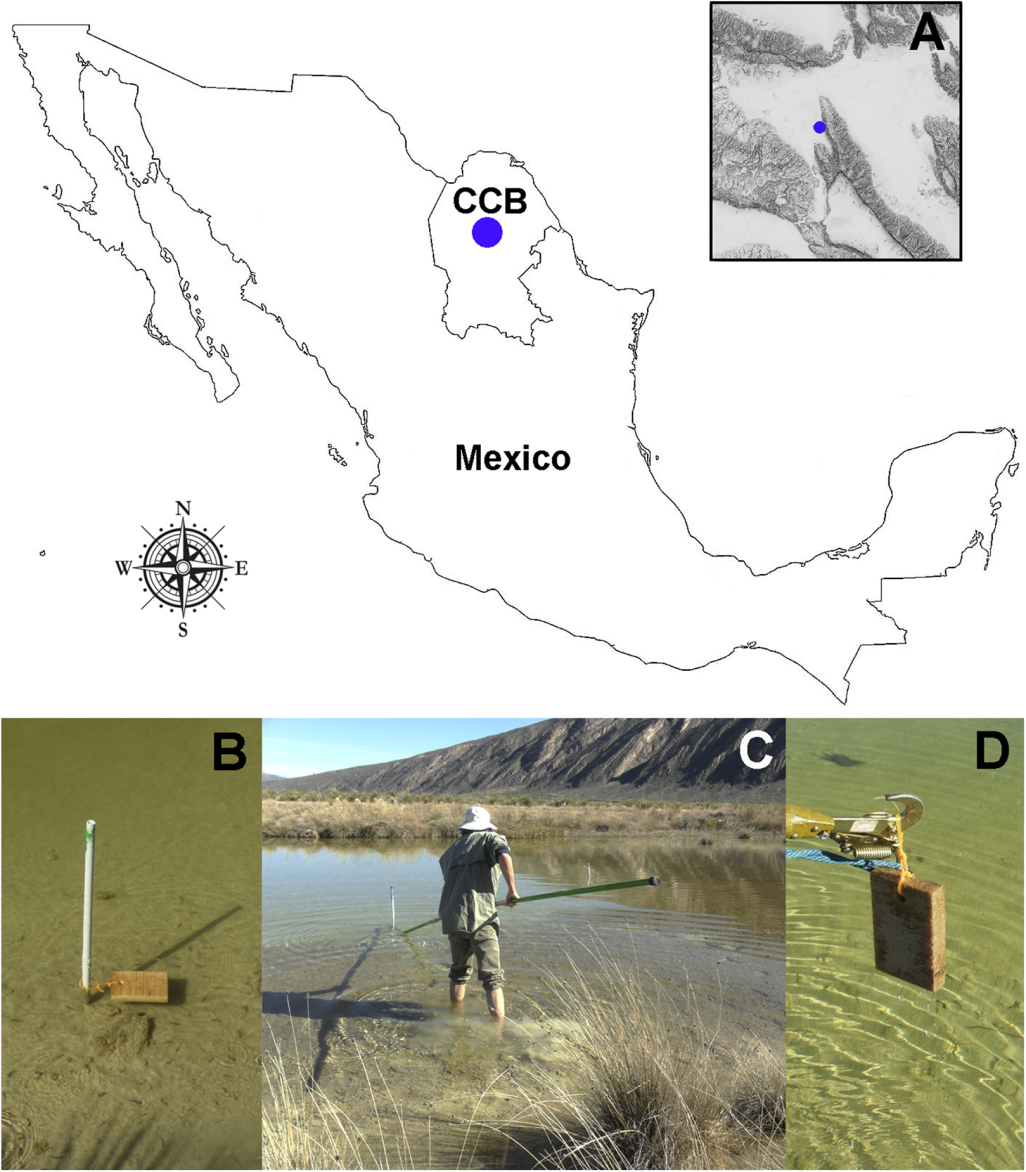
Study site and sampling procedures. (A) Detail on the geographic location of the Churince freshwater system within the CCB. (B) Set-up of the bating technique using *Pinus* sp. wood baits. (C and D) Recovery of the wood panels after six months of submersion. Photographer: P. Velez.

We submerged 15 wood baits of *Pinus* sp. (16 × 10 × 2 cm) along the Churince freshwater system (Figs. 1A and 1B) during the transition from wet to dry season, from September 2014 to March 2015 (field permit FAUT-0230, emitted by Secretaría de Medio Ambiente y Recursos Naturales, Subsecretaría de Gestión para la Protección Ambiental, Dirección de Vida Silvestre). An elevation map of the CCB (Fig. 1A) was generated using layers from the Instituto Nacional de Estadística y Geografía (INEGI, 2013) with the packages sp. (Pebesma & Bivand, 2005) and maptools (Bivand & Lewin-Koh, 2015). After six months, the wood panels were recovered (Figs. 1C and 1D), placed into individual Zip-lock® plastic bags, transported to the laboratory, and processed within 24 h of arrival to the laboratory. The test blocks were processed according to the methodology described by Jones (1971), which in summary consists of incubating test blocks in plastic boxes (with sterile moist paper towels forming a moist chamber). Moist chambers were kept at room temperature (22–26 °C) under natural daylight and examined periodically for the presence of mites and fungal structures over a period of six months using a stereo-microscope (Discovery V8 Stereo; Carl Zeiss, Göttingen, Germany).

### Microfungal isolation and identification

Fungi from wood baits were isolated using Potato Dextrose Agar (PDA; Fluka Analytical; Sigma-Aldrich, St. Louis, MO, USA) supplemented with 0.5 g/mL of penicillin-G and 0.3 g/mL of streptomycin sulfate to prevent bacterial development. The taxonomic assignment of axenic isolates was based on morphological and molecular characterization. The morphology of microfungal reproductive structures was examined (Samson et al., 2014; Yilmaz et al., 2016) using a transmitted light microscope “Carl Zeiss Axiostar Plus” equipped with the microscope software “Zeiss ZEN”. The universal DNA barcode marker for fungi (nuclear ribosomal internal transcribed spacer region, ITS rDNA; Schoch et al., 2012) was analyzed for each isolate. To do this, microfungi were transferred to Potato Dextrose liquid medium until adequate growth occurred. Mycelium was collected and DNA was extracted using the protocol described by Doyle & Doyle (1987). The ITS rDNA region was amplified and sequenced using ITS1 and ITS4 primers as previously described by White et al. (1990). Sanger sequencing reactions were performed in both directions by the High Throughput Genomics Center Facility, University of Washington and by the Macrogen Inc., South Korea. Isolates and total DNA were deposited in the culture collection of the Laboratorio de Evolución Molecular y Experimental, Instituto de Ecología, headed by Dr. Valeria Souza, and the Laboratorio de Ecología Molecular de Micromicetos en Ecosistemas Amenazados, Instituto de Biología, headed by Dr. Patricia Velez; both at the Universidad Nacional Autónoma de México (UNAM). Cultures and total DNA are available for research upon request.

Quality assessment and assembly of the sequences was performed using the finishing tool Consed version 29.0 (Ewing & Green, 1998; Ewing & Green, 1998; Gordon, Desmarais & Green, 2001). For the taxonomic assignment of microfungal isolates, sequence homology was evaluated through the comparison to the UNITE database version 7.2 using the BLAST algorithm (with an *e*-value > 0.001; Abarenkov et al., 2010; Koljalg et al., 2013). Sequence similarity for defining OTUs was set as proposed by Millberg, Boberg & Stenlid (2015), with a cut-off value of 98–100% for presumed species, 94–97% for genus level and 80–93% for order level. For conflicting hits, the lowest common rank level was used (Peršoh et al., 2010; Table S1).

### Mite culture, identification, and food preference bioassays

Oribatid mites were maintained on moist wood panels that were profusely colonized by the tested fungi until processing. For taxonomic identification and illustration, the specimens were mounted in lactic acid on temporary cavity slides. Images were obtained using an AxioCam MRC5 camera mounted on a Carl Zeiss AxioZoom V16 microscope. Mites were identified based on the taxonomic characteristics described for the genus *Trhypochthoniellus* (Balogh, 1972; Weigmann, 1999; Fujikawa, 2000; Kuriki, 2005).

Additionally, a phylogenetic reconstruction based on COI sequence data was performed. The gnatosome (bucal sections) and leg portions were dissected and collected into 0.2 mL PCR microtubes containing buffer (Phire Animal Tissue Direct PCR Kit; ThermoFisher Scientific; Waltham, MA, USA). The reactions were carried out according to the manufacturer’s protocol at a 55 °C annealing temperature. Oribatid specific primers were used (Arch1 and HCO2198; Heethoff et al., 2007) at a 0.5 μM final concentration. Negative controls were included, where the sterilized needle was dipped into the PCR buffer prior to mite dissection. Sanger sequencing reactions were performed in both directions by the Laboratorio de Secuenciación Genómica de la Biodiversidad y de la Salud, Instituto de Biología UNAM, México. The assembly of forward and reverse sequences was done using Geneious v.9.1 (Biomatters, Auckland, New Zeland). Sequences were manually edited and trimmed using BioEdit software (v7.0.5; Hall, 2005). Using a BLAST-n analysis, closely related NCBI sequences were selected to compute a phylogenetic reconstruction including analogous oribatid species and external taxonomic categories as previously reported by Klimov et al. (2018). Phylogenetic placement of the CCB mites was determined according to a Maximum-Likelihood (ML, 1,000 bootstrap) analysis run in RAxML v.8.2.10 (Stamatakis, Hoover & Rougemont, 2008) using GTRCAT model; *Cryptocellus* sp. AD1430 was defined as an outgroup. All ML inferences were performed in the CIPRES Science Gateway portal (Miller, Pfeiffer & Schwartz, 2010).

Mite diet preference experiments were implemented as described by Hubert et al. (2004). Oribatids were transferred from wood baits into culture chambers consisting of a 2 mm thick layer of plaster of Paris mixed with charcoal (ratio 9:1) in small Petri dishes (5 cm in diameter). Five individuals were placed in each culture chamber and starved for five days. Then, to corroborate active feeding, a fungal plug (1 cm^2^) of each one of the three isolated fungal taxa was offered to the mites individually. During a second experiment, food preference was tested by presenting fungi simultaneously to starved mites in the following combinations: *Pleosporales* sp. (a), *Talaromyces* sp. (b), and *Aspergillus niger* (c) individually; paired combinations a-b, a-c, and b-c; and simultaneously a, b, and c. Culture chambers were incubated under antiseptic conditions at 25 °C, 12 h light-12 h dark cycles, and 85% RH, and moistened regularly (3 day intervals) with 5 mL of sterile distilled water.

All experiments were run using negative controls (no fungus). Due to the limited availability of biological material on our wood panels, a replicate was implemented only for the first experiments (corroboration of the active consumption of a, b, and c). Individuals of each treatment were observed for several weeks (Table 1). During this period, the number of mites under, near, and far from the fungal plug was recorded. Also, the number of visits made by the mites among discs was counted. Based on this information, oribatids on discs were classified as “actively feeding,” while those which were walking around these discs were classified as “looking for food” (Hubert, Sustr & Smrz, 1999).

**Table 1:** Summary of food preference results.

Treatment	2 days		1 week		2 weeks	
	Experiment	Replicate	Experiment	Replicate	Experiment	Replicate
a	All mites meandered around the fungal plug searching for food	3 mites meandered around the fungal plug searching for food, 2 mites remained away from the feeding area	1 mite was actively feeding on the fungal plug, and 4 mites were searching for food around the fungal plug	All mites meandered around the fungal plug searching for food	3 mites were found dead, 2 mites meandered around the fungal plug	2 mites were found dead, 3 mites meandered around the fungal plug
b	All mites meandered around the fungal plug searching for food	All mites meandered around the fungal plug searching for food	All mites were actively feeding on the top of the fungal plug	4 mites were actively feeding on the top of the fungal plug, 1 mite was found dead	All mites visited the fungal plug, detection of 3 nymphs	All mites (4) visited the fungal plug
c	All mites visited the fungal plug searching for food	4 mites visited the fungal plug, and 1 was actively feeding	1 mite was feeding on the top of the fungal plug, 4 highly active mites meandered around the fungal plug	2 mites were actively feeding on the top of the fungal plug, 3 highly active mites meandered around the fungal plug	All mites were actively feeding on the top of the fungal plug, detection of 1 larvae and 3 nymphs	All mites were actively feeding on the top of the fungal plug, detection of 3 larvae and 3 nymphs
a-b	All the mites visited both fungal plugs, and remained searching for food in close proximity to the feeding area		2 mites visited a, and 3 mites visited b		1 mite visited a, and 3 mites visited b	
a-c	All mites visited c		1 mite visited a, and 4 mites visited c		1 mite meandered around a, 4 mites were actively feeding on the top of c, detection of 1 larvae and 2 nymphs	
b-c	2 mites visited b, and 3 mites visited c		2 mites visited b, 3 mites visited c		All mites were actively feeding on the top of c, detection of 3 larvae and 2 nymphs	
a-b-c	1 mite visited a, 1 mite visited b, and 3 mites visited c		2 mites meandering around b, and 3 mites actively feeding on c		1 mite visited b, 4 mites were actively feeding on the top of c, detection of 4 nymphs and 2 larvae	
Control	Mites with little activity	Mites with little activity	Mites with little activity	Mites with little activity	3 mites were found dead, and all died by the end of the fourth week	All mites were found dead

**Note:**

Treatments include controls (no microfungi) and several dietary options where microfungal taxa were presented individually and simultaneously to starved mites (*n* = 5). *Pleosporales* sp. (a), *Talaromyces* sp. (b), and *A. niger* (c).

### Gut-content analyses

Oribatids were examined for the content of their digestive tract through culture-dependent (plating) and culture-independent (direct PCR focusing on the ITS fungal barcoding region) methods. After four weeks of the food preference bioassays, two mites under each treatment were collected and superficially disinfected in 1.5 mL microtubes containing 1 mL solution of 75% methanol, and then transferred to 1.5 mL microtubes with 1 mL of sterile 0.03% Tween-80 solution. All tubes were gently vortexed for 30 s. Subsequently, under sterile conditions, the idiosome of each mite was dissected out with tungsten wire needles, squashed on concave slides, re-suspended in 300 μL of sterile water, and plated (100 μL) on Petri dishes containing PDA in triplicate per individual. To confirm the aseptic conditions during the experiment, negative controls were plated using 100 μL of sterile water.

Additionally, for the molecular analysis, the gut of superficially disinfected animals under each treatment was dissected out and collected into 0.2 mL PCR microtubes containing PCR buffer (Phire Animal Tissue Direct PCR Kit; ThermoFisher Scientific; Waltham, MA, USA). PCR reactions were performed according to the manufacturer’s protocol, using fungal-specific primers for the nuclear ribosomal ITS region (ITS1 and ITS4, 0.5uM final concentration). Two negative controls (buffer, and buffer where the sterilized dissecting needle was soaked) were also included. Sequencing reactions were carried out in both directions by the Laboratorio de Secuenciación Genómica de la Biodiversidad y de la Salud, Instituto de Biología UNAM, México. The quality assessment and sequences assembly were performed using the finishing tool Consed version 29.0 (Ewing & Green, 1998; Ewing et al., 1998; Gordon, Desmarais & Green, 2001). The taxonomic assignment for fungal gut-content sequences was based on the evaluation of sequence homology through the NCBI-BLAST algorithm (Table S1). The sequence similarity for taxonomic assignment was considered as proposed by Millberg, Boberg & Stenlid (2015).

## Results

**Figure 2:**
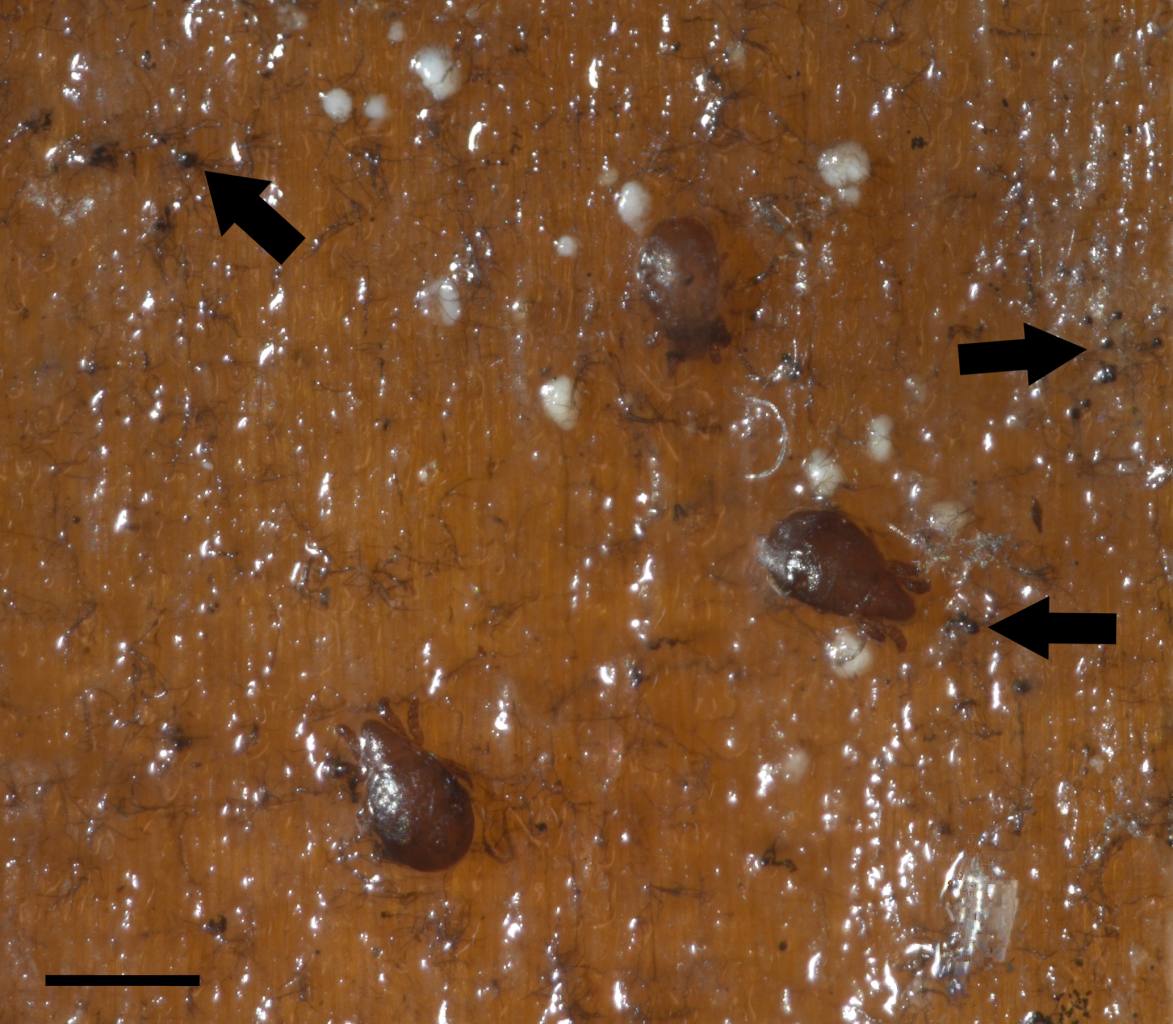
Co-occurring oribatid mites and fungi on *Pinus* sp. wood panels. Profusely developing fungal filamentous structures or hyphae (arrows). Bar = 500 μm. Photographer: M. Ojeda.

**Figure 3:**
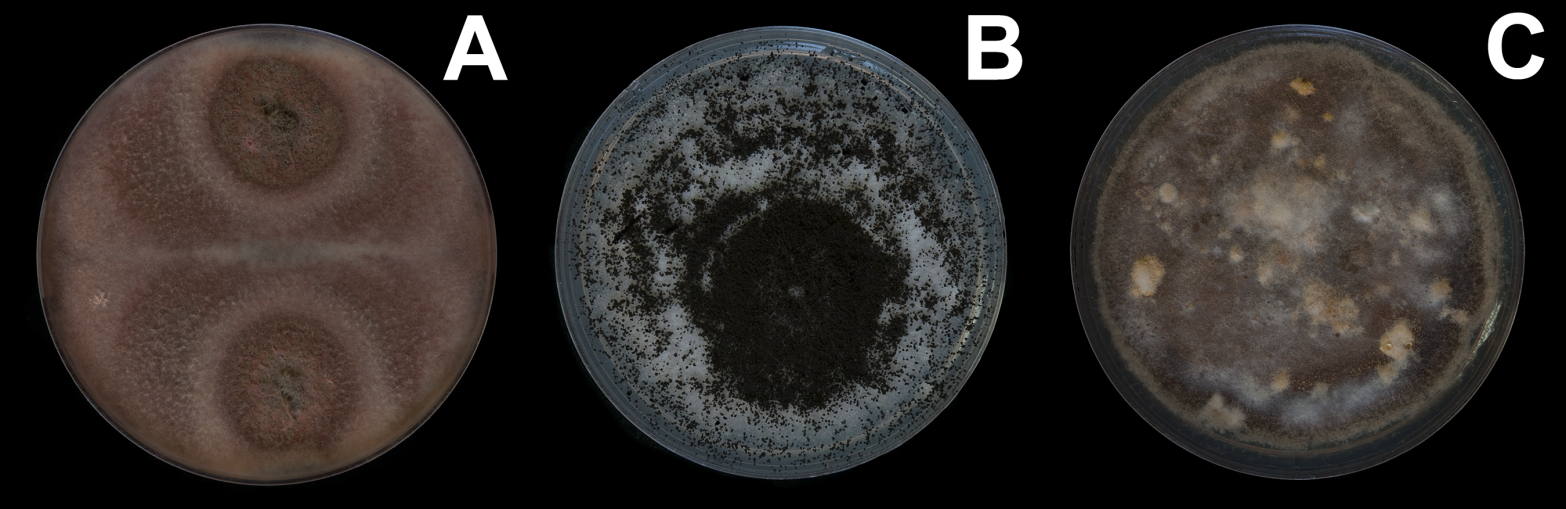
Fungal isolates tested during in vitro food preference bioassays. (A) *Pleosporales* sp. (B) *Aspergillus niger.* (C) *Talaromyces* sp. Photographer: C. Loyola.

**Figure 4:**
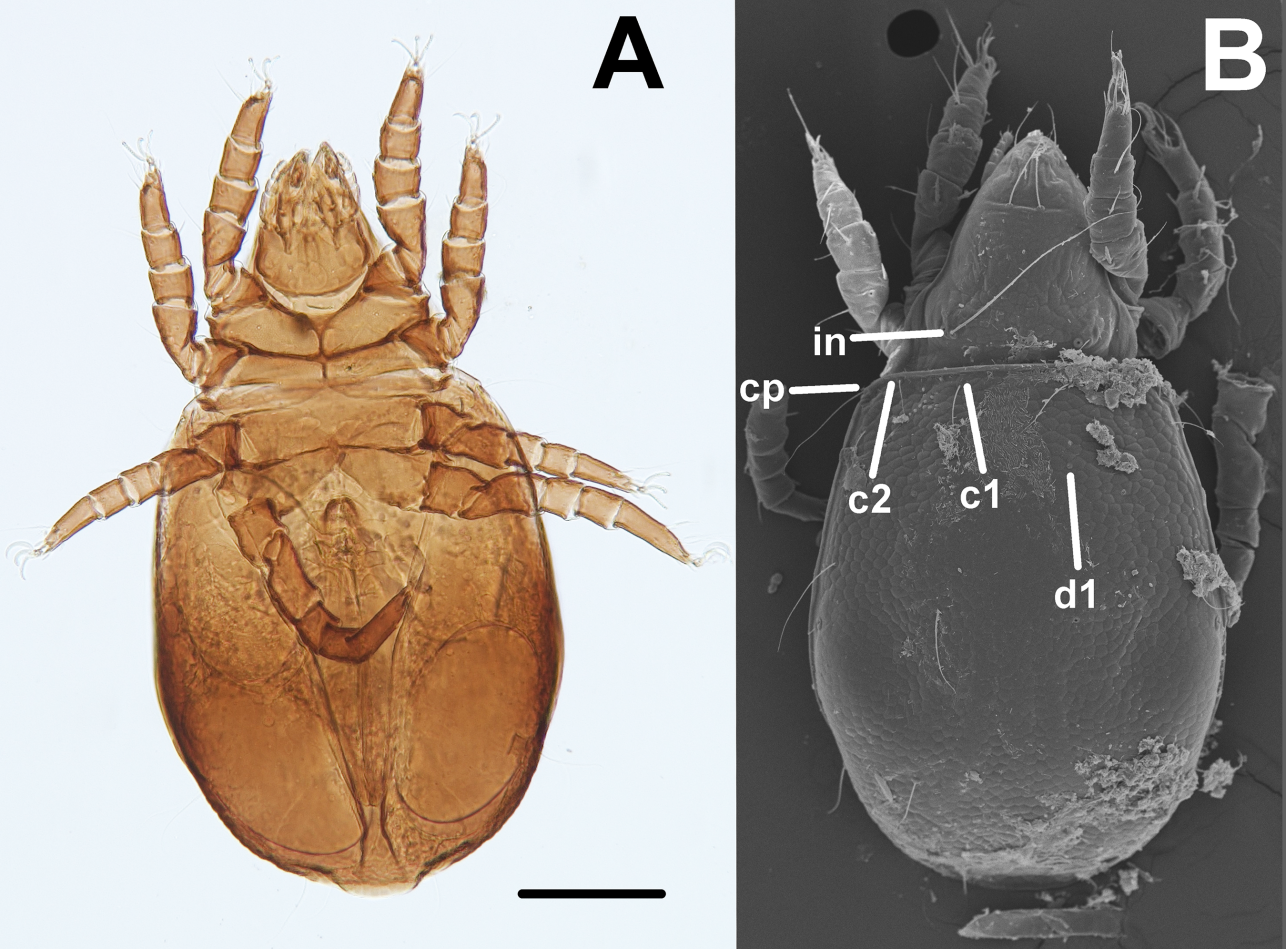
*Trhypochthoniellus* sp. habitus. (A) Ventral view. (B) Dorsal view, showing some general characteristics such as the absence of both botridial complex and sensilli. Bar = 100 μm. Photographer: M Ojeda.

**Figure 5:**
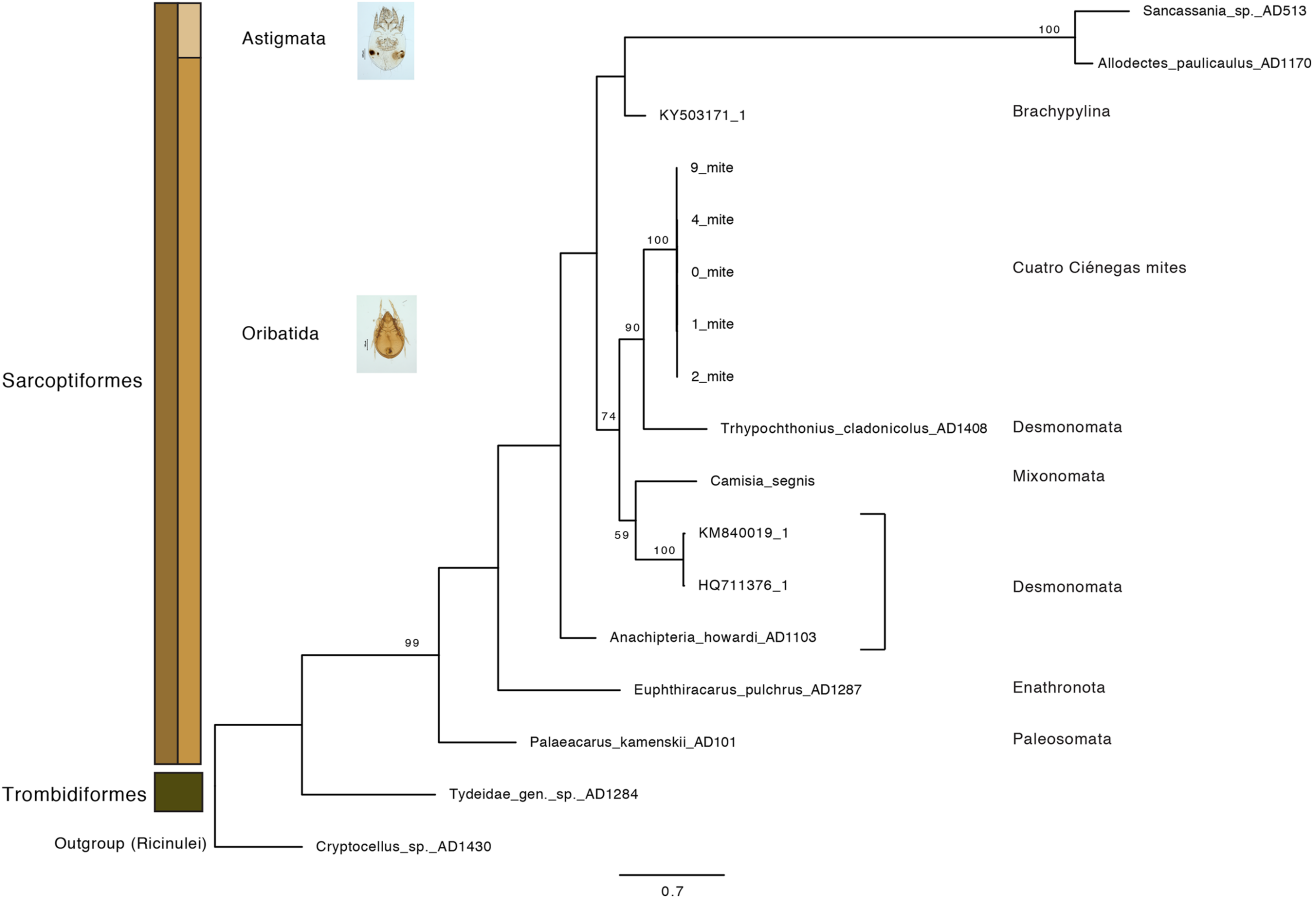
Maximum-Likelihood phylogeny of CCB mites inferred from COI sequences. The CCB oribatid mite sequences cluster together, forming a distant lineage from known *Trhypochthoniellus* oribatid taxa (Klimov et al., 2018 and NCBI retrieved sequences included). Bootstrap support for nodes with values higher than 50 percent are indicated. Photographer: M. Ojeda.

Oribatid mites were detected on test blocks where profuse fungal development was observed (Fig. 2). From these blocks, we isolated fungal taxa identified as *Pleosporales* sp., *Talaromyces* sp., and *Aspergillus niger* (Fig. 3; Table S1). Based on morphological and molecular characteristics, CCB mites were placed into the genus *Trhypochthoniellus* (Willmann, 1928), following basic trhypochthoniid characters as presented by Norton & Behan-Pelletier (2009) for Trhypochthoniidae. Morphologically, our oribatids differed from known *Trhypochthoniellus* taxa by: (1) the absence of both botridial complex and sensilli, (2) the number of genital setae, (3) rostrum shape, (4) general body size and (5) distance between notogastral setae c1–c1 compared with d1–d1, among some other characteristics (Fig. 4). This species is currently under the description process by M. Ojeda. Moreover, as reported for *T. longisetus*, CCB mites are parthenogenetic and no males were observed in the samples during experimental procedures. The ML phylogeny (Fig. 5) confirmed these observations clustering our mites within the same group, distant from most known taxa and with the closest affinity to Crotonioidea, represented by *Trhypochthonius cladonicola*.

Feeding preference experiments revealed that mites have varying dietary inclinations when the tested fungal taxa were offered simultaneously. Although, *Pleosporales* sp. and *Talaromyces* sp. were consumed by some individuals, the ubiquitous *A. niger* was consistently preferred in all treatments. After the first 48 h, mites in the three treatments were observed meandering around the fungal plug, yet in the treatment containing *A. niger* all the mites approached the fungus directly. For the following weeks, all mites in the treatment with *A. niger* were observed feeding actively over and under the fungal plug and the presences of larvae and nymphs was recorded. As for the other treatments, mites remained searching for food in close proximity to fungal plugs feeding occasionally. However, these were not detected over and/or under the fungal plug. Mites in the control plates died after four weeks (Table 1).

Gut content plating yielded no fungal development on any of our experimental and control Petri dishes. However, through the molecular gut-content analysis, we were able to recover genetic signatures corresponding to the fed *A. niger* and *Talaromyces* sp. Moreover, our findings indicated that when all the microfungi were offered simultaneously in food preference bioassays, the ITS sequence signal for *Talaromyces* sp. was detected. In paired combinations, *A. niger* was recovered over *Pleosporales* sp. and *Talaromyces* sp. (Table S1).

## Discussion

Our results suggest that CCB mites could represent a novel genetic lineage within *Trhypochthoniellus*, close to Crotonioidea, represented by *T. cladonicola*, yet further detailed taxonomic analyses are required to validate this hypothesis. The topology of our ML tree is equivalent to Klimov et al. (2018), and the value supporting the branch containing CCB mites within *Trhypochthoniellus* was high (90%). Remarkably, our mites grouped within a single phylogenetic cluster, diverging from known taxa, agreeing with diversity patterns (presence of unique lineages) reported for other organisms such as fish (Minckley, 1984), shrimp (Alvarez, Pedraza-Lara & Villalobos, 2014), and snails (Hershler, 1984) in the Churince system. In addition to this, the feeding habits of this novel CCB aquatic mite reinforce the idea of divergence though the occupation of a new niche space by feeding on a fungal species reported as unsuitable and unpalatable for other mites. Currently, the genus *Trhypochthoniellus* contains nine species; the type species (*Trhypochthoniellus longisetus* Berlese, 1904) with three cosmopolitan subspecies, seven species occurring in the Eastern Palaearctic and Oriental regions, and the recently described *T. chilensis* from mosses near a swamp in the Magallanean region of Chile (Ermilov & Weigmann, 2015).

Previous investigations in the Churince system revealed that freshwater microfungal communities are dominated by transient saprotrophic taxa which were inferred to be involved in the ecosystem’s functioning (Velez et al., 2016). Our mesocosms approach facilitated the recovery of interacting microbes, which were able to survive and reproduce under laboratory conditions, revealing their ecological niche. These wood baits demonstrated that transient aquatic saprotrophic microfungi represent a sustainable community in the Churince aquatic system, establishing trophic interactions with parthenogenetic aquatic oribatids. Herein, we demonstrated that these fungal saprobes fulfill a crucial role as part of food web. By sustaining and feeding aquatic oribatids, they participate in nutrient cycling. Since catalytic mechanisms have been associated with oribatid feeding and movement (Behan & Hill, 1978), a full understanding of their dietary habits is necessary in the CCB in order to elucidate their role in ecosystem energetics under oligotrophic conditions. Moreover, considering previous observations on the relationship between ample resource supply and the occurrence of parthenogenetic species (Fischer, Meyer & Maraun, 2014), our results suggest that aquatic transient microfungi provide plentiful nutrimental resources to sustain oribatid the development and growth of oribatid populations (presence of larvae and nymphs) in the CCB despite low nutrient conditions.

### Approximation to the niche space and dietary preferences in Trhypochthoniellus sp

In general, oribatids feed on a wide range of materials (Dhooria, 2016), having a dietary preference for microfungi with melanized cell walls (Mitchell & Parkinson, 1976; Maraun et al., 1998, 2003; Schneider & Maraun, 2005). In contrast, our results suggest that aquatic oribatids in the CCB may feed on non-dematiaceous taxa such as *A. niger, Pleosporales* sp. and *Talaromyces* sp. Though DNA may be degraded to some extent during gut passage (Renker et al., 2005), we were able to confirm these observations through the recovery of the fungal ITS genetic barcode region from the gut of our mites by direct PCR. Here it is important to mention that currently an inclusive genetic barcode for the Fungal Kingdom is still unapproachable. The best approximation has been achieved through the implementation of the ITS region as the standard fungal barcode. As this region has the highest probability of successful identification for the broadest range of fungi (Schoch et al., 2012). However, poor species-level resolution has been reported in certain taxonomic groups (e.g., Hughes, Tulloss & Petersen, 2018). Therefore, this limitation should be considered in ITS-based species delimitations.

Cuatro Ciénegas mites demonstrated their ability to feed on the usually avoided and unsuitable fungus *A. niger*, an apparently previously empty niche space for mites, according to previous investigations (Hubert et al., 2004). The consistent ingestion of *A. niger* in all the treatments of our study disagrees with previous findings classifying this species as non-palatable and unsuitable for mite development and population growth (Hartenstein, 1962; Sinha, 1966; Hubert et al., 2004). However, our results confirm other study findings where spores of this fungus have been detected in mite guts (Behan & Hill, 1978; Hubert, Kubátová & Šárová, 2000; Bandyopadhyay, Khatun & Chatterjee, 2009). In addition, we suggest that mite feeding on *Pleosporales* sp. may be associated with the wide distribution and abundance of this taxon in the CCB (Velez et al., 2016), representing a continuous and accessible food source. Similarly, the appeal of *A. niger* for our oribatids might be related to fast growth rates, abundant biomass and conidia production.

Even though multiple-choice tests may be biased by the spectrum of food items offered to individuals, the tested microfungi and mites were observed to coexist in our wood baits’ mesocosms (by definition a simplification of the environment), representing ecosystem dynamics. Hence, in order to delimit the total niche space of this oribatid, further experiments are needed evaluating of additional food sources, possible predators, and the mimicry of CCB distinctive physicochemical variables.

Identification of core factors associated with fungal palatability in microarthropods remains elusive (Koukol et al., 2009). Conventionally, feeding preferences have been related to fungal morphology and physiology. For example, mites avoid fast-growing microfungi, which may capture individuals in their long hyphae. Thus, species with short-hyphae are preferentially grazed (Mills & Sinha, 1971). Despite, the fact that *A. niger*, *Pleosporales* sp. and *Talaromyces* sp. may be considered as fast-growing taxa, our observations revealed that the mycelia of these microfungi allowed free movement of mite individuals on the wood panels and during in vitro bioassays, enabling active feeding.

**Figure 6:**
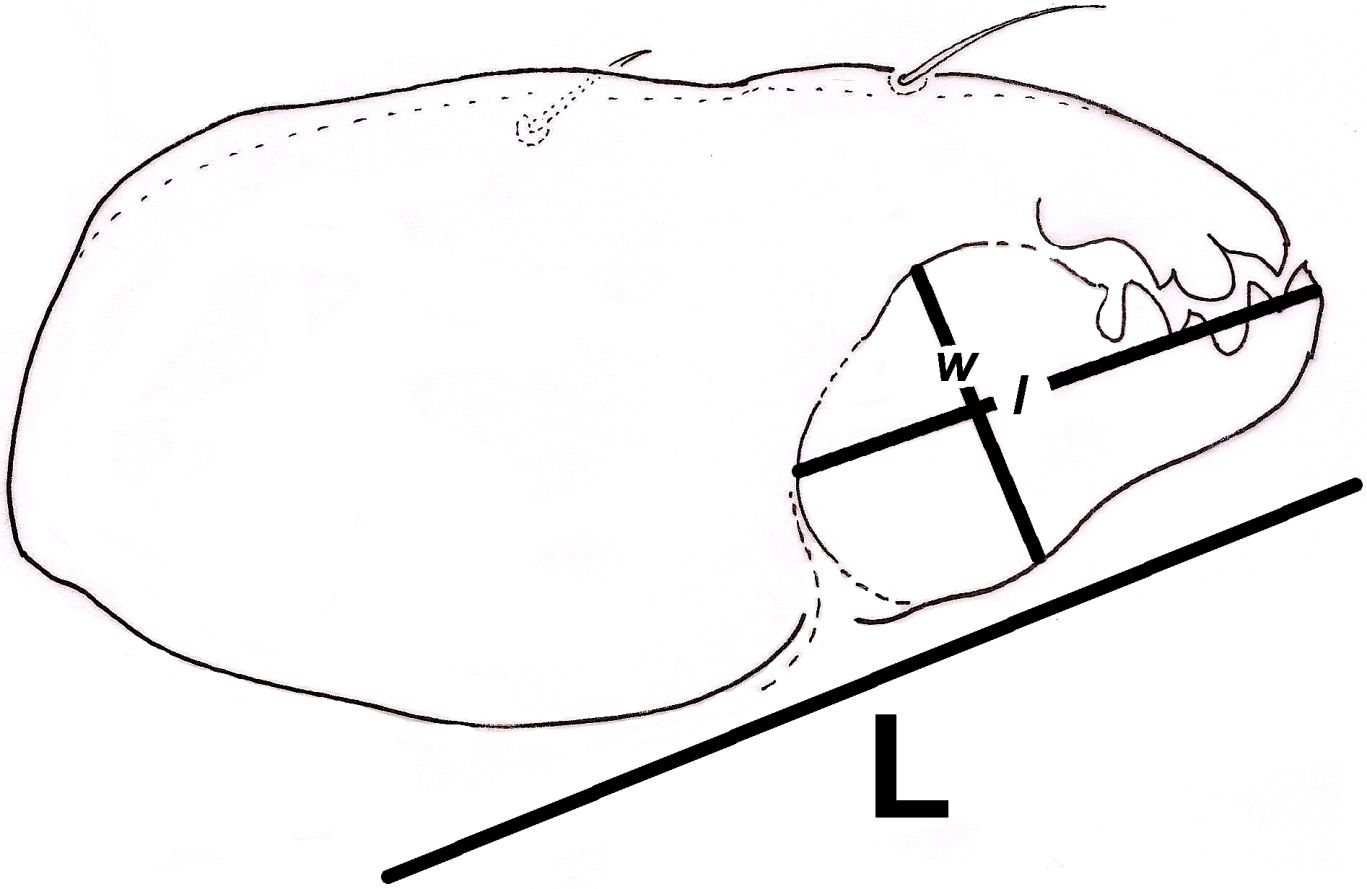
Relative width of *Digitus Mobilis* (RWD) approximation in *Trhypochthoniellus* sp. based on the shape of the infracapitulum, and its correlation with chelicera. L = length of chelicera; *l* = length of *digitus mobilis*; *w* = width of *digitus mobilis*.

Additionally, we approximated a Relative Width of Digitus Mobilis (RWD: relationship between length *l* and base width of the *digitus mobilis w*) of 4.5–5 μm in our oribatids (Fig. 6). This estimate is an indirect indicator of food items used by oribatid species (Kaneko, 1988), as mites with smaller RWD (around 2–4 μm) hypothetically feed selectively on minute matter (Wallwork, 1958). Therefore, conidia dimensions might be considered as a key trait for microarthropod ingestion (Koukol et al., 2009). This result suggests that spherical/ovoid, small (3–5 μm) conidia produced by fungal species in the present work are potentially consumable by mites, enabling the ingestion of all fungal components.

### Mite-mediated fungal dispersal

Fungal spore dispersal by animals (vertebrates and invertebrates) has been documented in some systems (Allen, 1987; Warner, Allen & MacMahon, 1987; Klironomos & Moutoglis, 1999; Gehring, Wolf & Theimer, 2002), yet information on microfungal dispersal agents remains scarce. Our culture-dependent results on the gut content analysis suggest that, based on their capabilities to digest fungal tissues (cell walls and contents), these oribatids may be considered more as grazers (Siepel & de Ruiter-Dijkman, 1993) than spore dispersers (via feces), as we could not recover fungal isolates from our culture-based approach. This agrees with previous work where thorough digestion of fungal cell content by mites is reported (Van der Drift, 1965; Ponge & Charpentie, 1981; Hubert, Zilova & Pekar, 2001), highlighting its alimentary value (Hubert et al., 2004).

Nevertheless, based on their fungal-based diet, the assessed mites may still represent important dispersers for transient aquatic fungi through some other means (Blackwell & Malloch, 1991; Renker, Alphei & Buscot, 2003; Rantalainen et al., 2004). Despite conidia dispersal via mite feces being unlikely, dispersal on the body surfaces of individuals may be significant (Wallwork, 1983; Bernini, 1986; Paine, Raffa & Harrington, 1997; Hubert, Kubátová & Šárová, 2000; Ocak, Dogan & Ayyildiz, 2008). As has been it was observed in other systems, this oribatid-aided conidia transport may represent an advantage for the colonization of new substrata, and faster recovery after disturbances under harsh, fluctuating environmental conditions inherent to the CCB (Hanlon & Anderson, 1979; Hanlon, 1981; Visser, Whittaker & Parkinson, 1981).

## Conclusion

The niche space created as a result of the trophic interactions between mites and microfungi is often overlooked or ignored despite the regulation of important community dynamics and ecosystem processes by these organisms (Warnock, Fitter & Usher, 1982; Finlay, 1985; Harris & Boerner, 1990; Gange & Brown, 1992). Herein, we introduce a novel genetic lineage of *Trhypochthoniellus* (Acari: Oribatida: Trhypochthoniidae) from a freshwater oligotrophic desert oasis in Mexico. The trophic niche space occupied by this mite is comprised of the ecological association with three abundant transient aquatic fungi including unpalatable taxa for other mite species, such as *A. niger*. The information demonstrated by this study is essential to understanding trophic interactions and their role shaping the niche space in low-nutrient ecosystems.

We suggest that our CCB oribatid mites are fungal grazers, demonstrating their key ecological role in energy turnover under oligotrophic conditions. This is particularly important as larger edaphic animals, such as earthworms, millipedes, and isopods (which usually play a major role in litter decomposition in temperate regions) are scarce in the CCB. We emphasize the need for further studies investigating the association between microarthropods and microfungi as this interaction affects microbial biomass and community structure, having potential widespread consequences in decomposition rates and nutrient cycling.

## Supplemental Information

10.7717/peerj.5200/supp-1 Supplemental Information 1 Table S1: Sequence information.

## Additional Information and Declarations

### Field Study Permissions

The following information was supplied relating to field study approvals (i.e., approving body and any reference numbers): Field experiments were approved by Secretaria de Medio Ambiente Recursos Naturales, Subsecretaría de Gestión para la Protección Ambiental, Dirección de Vida Silvestre (FAUT-0230).

### Data Availability

The following information was supplied regarding data availability: Sequences have been submitted to GenBank, and are reported in Table S1: KY548382, MH223590, MH223589, MH376285, MH376286, MH376287, MH376288, MH376289.
